# Association between serum sulfatide and carotid intima media thickness in patients with familial hypercholesterolemia

**DOI:** 10.1007/s10719-014-9555-5

**Published:** 2014-08-31

**Authors:** Gang Li, Rui Hu

**Affiliations:** 1 Cardiac Centre of Hebei General Hospital, Shijiazhuang, Hebei China; 2 Clinical Laboratory, The Second Hospital of Hebei Medical University, Shijiazhuang, Hebei China

**Keywords:** Serum sulfatide, Carotid intima–media thickness, Heterozygous familial hypercholes-terolemia

## Abstract

There is a positive association between sulfatide and atherosclerosis in an animal model for human familial hypercholesterolemia. Carotid intima–media thickness (IMT) is thought to be a marker of atherosclerosis in humans. We investigated the relationship between sulfatide and carotid IMT in heterozygous familial hypercholesterolemia (FH) patients. Thirty-five genetically-verified heterozygous patients with FH and 34 healthy controls were recruited into our study. We measured serum sulfatide levels, the carotid IMT, and conventional cardiovascular risk factors including obesity parameters, blood pressure, fasting blood glucose, and lipid profiles. Subjects with heterozygous FH had significantly elevated serum sulfatide, elevated total cholesterol, low-density lipoprotein cholesterol, and increased carotid IMT compared with control subjects. In patients with FH, univariate analysis showed that serum sulfatide was significantly correlated with carotid IMT. Multiple linear regression analysis indicated that serum sulfatide was the only independent predictor of carotid IMT in patients with FH. Patients with heterozygous FH had significantly higher carotid IMT and the level of serum sulfatide was independently associated with atherosclerotic progression. (R: 0.720, R^2^: 0.503, *p* < 0.001).

## Introduction

Sulfatides are esters of sulfuric acid with galactosylceramides at C3 of the galactosyl residue, that are widely distributed in various animal organs and sera, including humans [1]. Over the last two decades, Hara, *et al.* have been continuously reporting the possible involvement of sulfatides in the cardiovascular system. First, using an animal model for human FH-WHHL (Watanabe hereditable hyperlipidemic) rabbits, they did a series of experiments comparing normal and WHHL rabbits, and revealed that sulfatides, the major glycosphingolipids in serum lipoproteins, are markedly elevated in WHHL rabbits [2] and accumulated in atheromatous plaques in the aortae of WHHL rabbits [3], which suggested sulfatides maybe take part in the progress of atherosclerosis, even CVD (cardiovascular disease). We subsequently found a close correlation between low levels of serum sulfatides and a high risk of CVD in patients with end-stage renal failure. The findings suggested that sulfatides maybe a novel biomarker predicting the incidence of CVD in patients with end-stage renal failure [4]. We also found that sulfatides were associated with neointimal thickening after vascular injury [5]. In addition, it has been reported that exogenous sulfatide could cause the development of intimal hyperplasia [6]. Therefore, sulfatides may be possibly associated with atherosclerosis in humans.

Carotid intima–media thickness (IMT) measured by high-resolution ultrasonography is thought to be a marker of atherosclerosis that is associated with future cardiovascular events [7, 8].

In this study, we investigated the association between sulfatide levels and carotid IMT in FH subjects who had no major cardiovascular risk factors except hypercholesterolemia, in order to confirm the possible association between sulfatides and atherosclerosis.

## Method

### Subjects

We recruited 35 patients with low density lipoprotein cholesterol (LDL-C) >4.92 mmol/l and positive family history of hypercholesterolemia in a genetic screening program for FH in Shijiazhuang, China, from October 2008 to November 2010. Charng’s methodology was used in the subjects for mutation detection of FH [9]. Other secondary causes of hypercholesterolemia were excluded, including nephrotic syndrome, liver disease, hypothyroidism, and diabetes mellitus. We also recruited 34 healthy people as the control group. These healthy individual’s LDL-C levels were less than 3.37 mmol/l and their family members were genetically verified as not carrying an FH-related mutation allele.

This study was conducted in accordance with the Declaration of Helsinki. Signed informed consent was obtained from all of the subjects, and the Medical Ethics Committee of the HeBei General Hospital approved the study protocols.

### Clinical characteristics and biochemical analysis

By a standardized questionnaire, trained nurses interviewed all participants and obtained their medical history. Participants rested for 10 min prior to their blood pressure being checked twice at an interval of at least 1 min. The mean value of these two measurements was used for the analyses. Each participant was clothed only in a light gown and their weight and height were measured. We calculated their body mass index as body weight (kg) divided by the square of height (m^2^). Subsequently, the same examiner measured waist circumference between the lowest rib and the iliac crest in a standing position.

All medications that could affect lipid levels were discontinued 1 month previous to the study. After an overnight fast of 12 to 14 h, blood samples were obtained from all subjects early in the morning. Total cholesterol, high-density lipoprotein (HDL) cholesterol, triglyceride levels, and the low-density lipoprotein (LDL) cholesterol level in blood samples were measured by an autobiochemical analysis system (AU2700, Olympus, Japan). The concentrations of apolipoprotein A1 and apolipoprotein B were measured by nephelometry (Behring Diagnostic, Marburg, Germany). The cholesterol-year score was calculated with the following equation: cholesterol-year score = yearly mean cholesterol serum level × age. For the measurement of the sulfatides, the serum was stored at −80 °C until analyzed.

### Measurement of serum sulfatide

Sulfatides were extracted from specimens using a hexane–isopropanol mixture and analyzed as lyso-forms (sulfatides without fatty acids) using a high-throughput method developed in our laboratory [10]. Briefly, the total lipids were extracted from 50 μl of serum with n-hexane: isopropanol (3:2, v/v). After dried and hydrolyzed with 0.1 N NaOH in 90 % methanol at 150 °C for 30 min, sulfatides were converted to lyso-sulfatides. Subsequently, samples were desalted by Mono-tip C18 tips (GL Sciences, Tokyo, Japan) and analyzed by matrix-assisted laser desorption ionization time-of-flight mass spectrometry with delayed ion extraction using a Voyager Elite XL Biospectrometry Workstation (PerSeptive Biosystems, Framingham, MA, USA). A nitrogen laser (337 nm) was used for ionization and negative ion mode detection was employed.

### Measurement of carotid intima–media thickness

The carotid arteries were evaluated by a single operator, blind to subject details with a high-resolution B-mode ultrasonography instrument using a 7.5 MHz probe (SSA-270A; Toshiba, Tokyo, Japan). Participants were scanned in the supine position with the neck hyperextended. Both common carotid arteries were thoroughly scanned from proximal to distal to the bifurcation. Frozen photocopies of end-diastolic images in the longitudinal view showed the bifurcation were captured. All images were taken when the inner echoes of both near and far wall were clearly visible. The IMT was measured at the far wall of both of the common carotid arteries about 1 cm proximal to the carotid bulb and defined as the mean of the maximal IMT of each common carotid artery.

### Statistical analysis

Results are shown by means and standard deviations (SDs) for continuous variables and proportions for categorical variables. T-tests were used in between-group comparisons. In FH subjects, Spearman’s correlation coefficients were determined to assess the association between continuous carotid IMT, conventional plasma lipids and lipoproteins, blood pressure values, and serum sulfatide levels. In order to test whether these variables had independent effects on the carotid IMT of FH subjects, a stepwise multiple linear regression analysis was performed and the predictors for carotid IMT were determined.

The SPSS 13.0 program (SPSS Inc., Chicago, IL, USA) was used to perform all statistical analyses. All tests were 2-sided and a value of *p* < 0.05 was considered statistically significant.

## Results

**Fig. 1:**
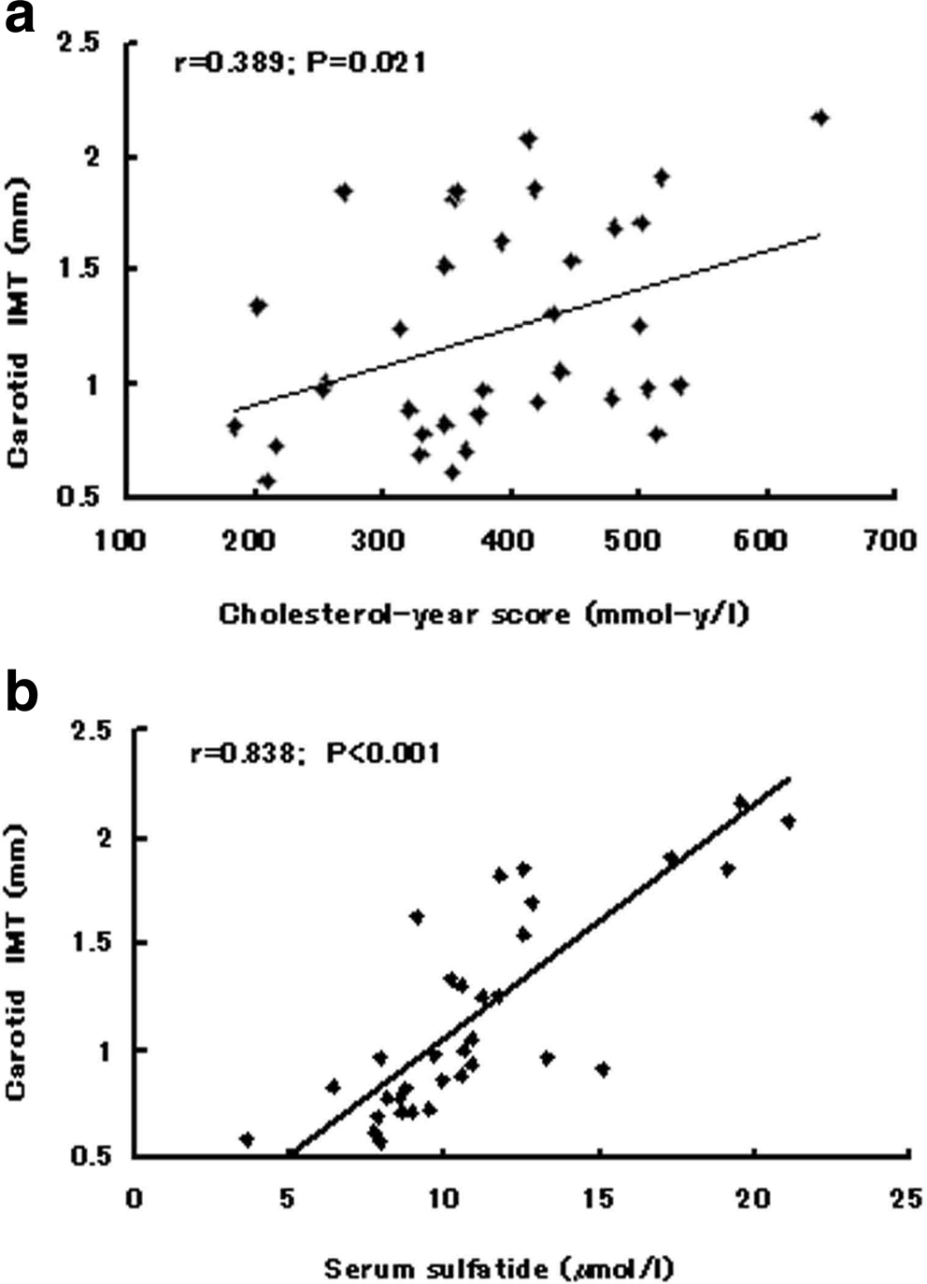
**a** Graph shows correlation between cholesterol-year score and carotid IMT (*r* = 0.389, *p* < 0.05). **b** Graph shows correlation between serum sulfatide and carotid IMT (*r* = 0.838, *p* < 0.001)

The baseline characteristics of the study subjects are presented in Table 1. The subjects with either a history of cardiovascular disease, hypertension, or diabetes mellitus were excluded in our study. Five of the FH subjects and three of the control subjects were current smokers. Compared with the control group, FH subjects had significantly elevated serum levels of total cholesterol, LDL cholesterol, and apolipoprotein B. In addition, the cholesterol-year score, serum sulfatide levels, and carotid IMT in FH subjects, were significantly higher than those of the control subjects (Table 1). As we all known, the calculated cholesterol-year score is often thought a marker of total cholesterol burden, and carotid IMT is often thought a marker of systemic atherosclerosis. In our study, as shown in Fig. 1a, the cholesterol-year score was positively correlated with the carotid IMT (*r* = 0.389, *p* < 0.05), which is consistent with the findings in a previous study [11]. In addition, the serum sulfatide levels were also positively correlated with the carotid IMT (*r* = 0.838, *p* < 0.001) (Fig. 1b). The correlation between serum sulfatide levels and carotid IMT seems to be closer than the correlation between cholesterol-year score and carotid IMT.

**Table 1:** Baseline and clinical data of controls and individuals with familial hypercholesterolemia

	Control (*n* = 34)	Familial hypercholesterol subjects (*n* = 35)	Significance
Age (years)	48.1 ± 11.6	48.4 ± 11.3	NS
Gender (male)(%)	50.0	48.6	NS
BMI (kg/m^2^)	24.2 ± 1.8	24.7 ± 1.7	NS
Waist circumference (cm)	85.0 ± 5.4	83.8 ± 5.2	NS
Waist-hip ratio	0.86 ± 0.04	0.86 ± 0.04	NS
SBP (mmHg)	130 ± 14	136 ± 12	NS
DBP (mmHg)	79 ± 12	82 ± 10	NS
Fasting blood glucose (mmol/l)	5.7 ± 0.9	5.4 ± 0.6	NS
Total cholesterol (mmol/l)	5.1 ± 0.6	7.9 ± 1.2	<0.001
Triglyceride (mmol/l)	1.4 ± 0.5	1.5 ± 0.5	NS
HDL-C (mmol/l)	1.3 ± 0.3	1.3 ± 0.3	NS
LDL-C (mmol/l)	3.1 ± 0.7	5.5 ± 1.0	<0.001
Cholesterol-year score (mmol-y/l)	247.1 ± 71.8	381.8 ± 105.9	<0.001
Apolipoprotein B (g/l)	1.0 ± 0.2	1.4 ± 0.3	<0.001
Apolipoprotein A1 (g/l)	1.3 ± 0.1	1.3 ± 0.1	NS
Serum sulfatide (μmol/l)	8.14 ± 2.68	11.30 ± 3.91	<0.001
Carotid IMT (mm)	0.77 ± 0.13	1.22 ± 0.52	<0.001

*NS* Not significant; *BMI* Body mass index; *SBP* Systolic blood pressure; *DBP* Diastolic blood pressure; *HDL-C* High density lipoprotein- cholesterol; *LDL-C* Low-density lipoprotein- cholesterol; *IMT* Intima media thickness

Table 2 showed that the carotid IMT was significantly correlated with age, body mass index, waist circumference, waist-hip ratio, systolic and diastolic blood pressures, triglyceride levels, apolipoprotein B levels, the cholesterol-year score, and serum sulfatide level. In addition, the serum sulfatide level was also associated with age, body mass index, waist circumference, waist-hip ratio, systolic and diastolic blood pressures, triglyceride levels, apolipoprotein B levels, and the cholesterol-year score.

**Table 2:** Spearman’s correlation coefficients for risk factors in individuals with familial hypercholesterolemia

	Carotid intima media thickness		Serum sulfatide	
	*r*	*P* value	*r*	*P* value
Age	0.436	<0.001	0.402	<0.001
Sex	−0.031	NS	−0.056	NS
BMI	0.632	<0.05	0.695	<0.05
Waist circumference	0.600	<0.05	0.725	<0.05
Waist-hip ratio	0.678	<0.05	0.726	<0.05
SBP	0.401	<0.05	0.505	<0.05
DBP	0.412	<0.05	0.591	<0.05
Fasting blood glucose	0.418	NS	0.414	NS
Total cholesterol	0.281	NS	0.216	NS
Triglyceride	0.512	<0.05	0.101	<0.05
HDL-C	−0.181	NS	−0.116	NS
LDL-C	0.284	NS	0.316	NS
Cholesterol-year score	0.526	<0.001	0.407	<0.001
Apolipoprotein B	0.384	<0.05	0.516	<0.05
Apolipoprotein A1	0.054	NS	0.026	NS
Serum sulfatide	0.600	<0.001	–	–

*NS* Not significant; *BMI* Body mass index; *SBP* Systolic blood pressure; *DBP* Diastolic blood pressure; *HDL-C* High density lipoprotein- cholesterol; *LDL-C* Low-density lipoprotein- cholesterol

We further divided FH subjects into 2 groups according to the extent of carotid IMT (cut-off value: 1 mm). Listed in Table 3, FH subjects with a carotid IMT > 1 mm were older. The body mass index, waist circumference, triglyceride levels, and the cholesterol-year score in FH subjects with a carotid IMT > 1 mm were higher than those in FH subjects with IMT ≤ 1 mm. In addition, the serum sulfatide level in FH subjects with a carotid IMT > 1 mm was significantly higher than that in FH subjects with IMT ≤ 1 mm. In order to clarify the correlation of serum sulfatide and carotid IMT, we performed stepwise multiple linear regression analyses for serum sulfatide and carotid IMT, adjusted for age, gender, body mass index, waist circumference, systolic and diastolic blood pressures, triglyceride levels, apolipoprotein B levels, fasting blood glucose, and the cholesterol-year score. The results showed that the serum sulfatide level was an independent predictor for the extent of carotid IMT (Partial R: 0.720, R^2^: 0.503, *p* < 0.001).

**Table 3:** Basic characteristics in individuals with familial hypercholesterolemia grouped by carotid intima media thickness

	Carotid intima media thickness ≤1 mm (*n* = 17)	Carotid intima media thickness >1 mm (*n* = 18)	Significance
Age (years)	41.8 ± 11.6	54.6 ± 14.2	<0.05
Gender (male)(%)	47.8	49.1	NS
BMI (kg/m^2^)	23.6 ± 3.1	25.7 ± 2.6	<0.05
Waist circumference (cm)	81.4 ± 6.0	86.0 ± 7.3	<0.05
SBP (mmHg)	136 ± 13	136 ± 11	NS
DBP (mmHg)	84 ± 12	81 ± 8	NS
Fasting blood glucose (mmol/l)	5.4 ± 0.5	5.4 ± 0.6	NS
Total cholesterol (mmol/l)	7.8 ± 1.3	7.9 ± 1.2	NS
Triglyceride (mmol/l)	1.3 ± 0.4	1.7 ± 0.6	<0.05
HDL-C (mmol/l)	1.2 ± 0.2	1.3 ± 0.3	NS
LDL-C (mmol/l)	5.6 ± 0.9	5.4 ± 1.1	NS
Cholesterol-year score (mmol-y/l)	325.5 ± 94.8	437.7 ± 148.5	<0.05
Apolipoprotein B (g/l)	1.3 ± 0.3	1.4 ± 0.3	NS
Apolipoprotein A1 (g/l)	1.3 ± 0.2	1.3 ± 0.1	NS
Serum sulfatide (μmol/l)	9.08 ± 2.57	13.40 ± 3.96	<0.001

*NS* Not significant; *BMI* Body mass index; *SBP* Systolic blood pressure; *DBP* Diastolic blood pressure; *HDL-C* High density lipoprotein- cholesterol; *LDL-C* Low-density lipoprotein- cholesterol; *IMT* Intima media thickness

## Discussion

The carotid intima media thickness is a good surrogate marker for atherosclerosis and is commonly used to measure the extent of atherosclerosis [12, 13]. In this study, we have found that serum sulfatide level is significantly and independently associated with increased IMT in familial hypercholesterolemia patients, even after controlling for other atherosclerosis risk factors. Therefore, serum sulfatide may be a novel risk factor for atherosclerosis.

As we all know, low-grade inflammation has been implicated in the progression of atherosclerosis [14]. One previous study has demonstrated a role for sulfatide in atherogenesis and vascular inflammation [5].

Firstly, sulfatide exists in serum lipoprotein of various mammals including humans [10]. Studies revealed that the increased sulfatide in both lipoprotein and atherosclerotic plaques in WHHL rabbits- an animal model for human FH, is intimately correlated with the development of atherosclerosis: in serum lipoproteins of WHHL rabbits, sulfatide content (121 nmol/ml serum) is markedly increased by 40-fold over the normal level, while phospholipid content is about 7 times the normal level due to hyperlipidemia. The level of sulfatide is sufficiently high to prolong the fibrin-precipitation time to twice the normal level and the enrichment of sulfatide in serum lipoprotein is correlated to the self-protecting effect against thrombosis by prolongation of the blood-coagulation time. Furthermore, lipid analysis of atherosclerotic aortae of WHHL rabbits revealed that a large amount of sulfatide was accumulated there, while normal aorta contained no sulfatide at all [2, 3]. We also found that a large proportion of all serum sulfatide is derived from the liver and that the remainder is derived from the small intestine [15]. Redundant circulating sulfatides synthesized in the liver and small intestine may be accumulated in atherosclerotic plaques in FH.

Secondly, sulfatide was demonstrated to play an important physiological role in the vascular inflammation associated with the development of atherothrombosis or atherosclerosis [14]. In the vascular inflammatory process, activation of leukocytes, neutrophils as well as monocytes, and their interactions with platelets are mediated by cell adhesion molecules. The cross talk of leukocyte integrin Mac-1 (CD11b/CD18) and platelet membrane surface P-selectin is known to play an especially important role [16, 17]. The activation and up-regulation of Mac-1 on the surface of neutrophils and an increase in the expression of P-selectin on the surface of platelets were associated with the increased vascular wall thickness [18]. Sulfatides are known to be a native ligand of P-selectin and could interact with multiple cell adhesion molecules [19]. Sulfatides are also expressed on the membrane surface of, and excreted by, neutrophils [20]. Since neutrophil activation occurred after vascular inflammation [21], sulfatides released from activated neutrophils may also act agonistically as a P-selectin ligand and promote P-selectin-Mac-1 cross talk, resulting in the activation of Mac-1 (*i.e.*, the reactivation of neutrophils), subsequently, resulting in vascular wall thickness and lumen loss [18, 21].

Thirdly, we should point out some of the limitations of our study: the relatively small sample size could not reflect the association between serum sulfatide and carotid IMT in human FH completely.

At last, we showed serum sulfatide levels are significantly associated with increased carotid IMT, namely with the progress of atherosclerosis, in FH patients. Therefore, the sulfatide-dependent pathway would be a novel target for prevention and/or treatment of atherosclerosis.
